# The effect of Mass Drug Administration for trachoma on antibodies to *Chlamydia trachomatis* pgp3 in children

**DOI:** 10.1038/s41598-020-71833-x

**Published:** 2020-09-16

**Authors:** Sheila K. West, Beatriz Munoz, Harran Mkocha, Charlotte A. Gaydos, Thomas C. Quinn

**Affiliations:** 1 Dana Center for Preventive Ophthalmology, Wilmer Eye Institute, Johns Hopkins School of Medicine, Woods Room 155, 600 N Wolfe St, Baltimore, MD 21205 USA; 2 International Chlamydia Laboratory, Department of Medicine, Johns Hopkins University, Baltimore, MD USA; 3 Kongwa Trachoma Project, Kongwa, Tanzania; 4 National Institute for Allergy and Infectious Diseases, National Institutes of Health, Bethesda, MD USA

**Keywords:** Medical research, Risk factors

## Abstract

A serologic test for antibodies to chlamydia may be a useful tool for trachoma surveillance. However, little is known about the longitudinal stability of antibody status, especially following Mass Drug Administration (MDA), which is critical to understanding serostatus in trachoma-endemic areas. A longitudinal cohort of 1908 children ages 1–9 years in Tanzania from 50 communities were followed at baseline and for 6 months after MDA. They were evaluated for clinical trachoma, conjunctival swabs were tested for chlamydial infection using GeneXpert platform, and blood spots were collected on filter paper and dried to test for antibodies to Chlamydia trachomatis pgp3 using the Luminex platform. 6.3% of children in the study had infection, and coverage with MDA was 97%. 670 (35%) were sero-positive for pgp3 antibodies at baseline, and 4.0% of these seroreverted to negative following MDA. Of those seronegative at baseline, 3.6% seroconverted. The individual change in log median fluorescence intensity(MFI-BG) values was -0.15 overall (p < .001). Seroconversion rates were lower following MDA and seroreversion rates were slightly higher compared to rates in this same cohort in the absence of MDA. MDA has a small effect on reduction of MFI-BG.

## Introduction

Trachoma, a chronic conjunctivitis caused by *C. trachomatis*, is still the leading infectious cause of blindness world-wide, although strides towards elimination are being achieved^1^. The World Health Organization (WHO) has set a goal of elimination of blinding trachoma as a public health problem by the year 2020^2^. For trachoma, the elimination goal is a prevalence at district level of trachomatous inflammation-follicular (TF) of < 5% in children ages 1–9 years. As more endemic districts within countries strive to achieve the elimination goal, they face the task of program impact assessment surveys and surveillance surveys for potential re-emergence. Currently, the WHO recommends that impact assessments be carried out following between 1–5 rounds of MDA (depending on the baseline prevalence of TF), and at least two years after cessation of mass drug administration, a surveillance prevalence survey for TF be undertaken in the district to re-confirm < 5% level of TF^3^.

However, grading TF in the field when the prevalence is low has generated cause for concern. Data from villages where TF is above 5% (but still low) in the absence of infection with *C. trachomatis* have been reported, raising concerns for over grading^4,5^. At the same time, districts where trachoma is less than 5% have also reported the presence of infection^6^ at the time of surveillance, suggesting that some level of infection could be tolerated and disease does not re-emerge.

When assessed cross sectionally in surveys, both TF and infection provide snapshots of the current prevalence but provide limited information about ongoing transmission or risk of re-emergence. For this reason, further work on additional surveillance tools that may provide more information has been recommended^3^. One potential tool, which has shown promise, is the use of a test for antibodies to Chlamydial antigen pgp3. Serology would be particularly useful if it reflects cumulative exposure to trachoma, thus allowing the interpretation that low or absent seropositivity reflects the absence of ongoing transmission^7^. We have previously shown that in the absence of MDA, where trachoma prevalence is < 10%, sero-reversion is around 6.4% per year, and seroconversion is around 10% per year^8^. However, the effect of MDA on serologic status in areas where trachoma is > 5% but low is unknown. Previous research in a hyper-endemic community has shown that chlamydia antibody seropositivity remains high, even after mass drug treatment (MDA), with no seroreversion six months after MDA; however, a test for antibodies is not likely to be used for impact assessment in the context of a hyperendemic area^9^.

While MDA lowers the community pool of infection and thus an effect on seroconversion rates is expected, if MDA also affects rates of seroreversion then the usefulness of serostatus as a marker of cumulative exposure to trachoma may be more complex than originally thought. In this study, we have undertaken a longitudinal study of trachoma, infection, and serologic status of children age 1–9 years pre- and 6 months post- MDA in 50 communities where trachoma was formerly hyper-endemic.

## Methods

### Population

Kongwa district in Tanzania was a formerly trachoma hyperendemic area whose prevalence of trachoma decreased to < 10% by 2013^10^. In April-June 2015, a random sample of 51 children ages 1–9 years in each of 50 communities that were enrolled in a clinical trial of surveillance strategies^10^ was selected for a longitudinal cohort study of the change in antibody status over time. Random selection of children was based on a complete census of all residents of the communities, and included age and gender of each resident. The baseline survey for this study was conducted in November 2015 followed by MDA for all residents of the district in April 2016. In October, 2016 we followed up the cohort to determine the change in infection, trachoma, and antibody status over time.

### Survey

A trained trachoma grader, using a flashlight and 2·5 loupes, assessed each eyelid for the presence or absence of trachomatous inflammation-follicular (TF) and trachomatous inflammation-intense (TI) using the World Health Organization simplified grading scheme^11^. An ocular photograph, taken of the right eye of every 5th child plus all children with trachoma, ensured at least 50 photographs for purposes of monitoring drift in grading over time. A handheld Nikon D-series camera (D-40) with a 105 mm f/2·8D AF Macro Nikkor Autofocus Lens was used^8^.

Following a strict protocol to avoid field contamination, at baseline a swab was taken of the left eye of every child, stored dry in a refrigerator for up to 30 days, shipped to Johns Hopkins University, where it was stored at − 80 °C until processed. In addition, a negative field control ("blue air swab") was taken on a randomly chosen 5% sample to monitor contamination. For each negative field control, the examiner passed a sterile Dacron swab within 1 inch of the individual’s conjunctiva, and these were labeled and processed identically to true conjunctival swabs. The laboratory personnel were masked to field control and regular swabs^8^.

Blood was taken from a single finger prick, filled up to six circular extensions which were calibrated to hold 10 µl of blood. The blood spots were dried, stored in a freezer until shipped to the International Chlamydia Laboratory at Johns Hopkins and processed for antibodies to pgp3 on Luminex 100 platform^7^.

### Mass Drug Administration

Mass Drug Administration with a single dose of azithromycin, height based dosing using height sticks with a target of 20 mg/kg, was provided to all communities of Kongwa District by the Tanzania National Trachoma program. They had their own census of the communities, and provided MDA over the course of a few days. We kept our own records of coverage for the study children, and went back to enhance coverage for the study children to ensure they received azithromycin. We also targeted 20 mg/kg, but weighed children under age 2 years and those children not yet able to stand, and provided suspension for them at the proper weight-based dose. We used height sticks for those age 2 and older and dosed them according to the suspension guidelines or tablet guidelines on the sticks used by the national program. In addition, using our own census data, we did post MDA coverage surveys in the 50 communities using a random sample of at least 30 households in each village to estimate actual coverage in the communities, both of the total population and of the children ages 1–9. MDA was supposed to occur directly after the baseline survey in November 2015, but was delayed until April 2016.

### Laboratory processing

The ocular swabs were processed using a published pooling strategy^12,13^ (4–5 per pool) within 90 days using the APTIMA Combo 2 (AC2) (Hologic Inc., San Diego CA) commercial test for *C. trachomatis*. The pool results were recorded as positive or negative for *C. trachomatis*, or invalid. For pools that yielded a negative result, all specimens in that pool were considered to be negative for *C. trachomatis*. For each pool that yielded a positive result, the original samples were retested in order to determine which sample(s) had a positive result. Invalid pools occur when the test result yields a parameter outside the normal expected ranges (e.g., 0 or > 4,500). The pool is retested as well as the individual samples contained in that pool, to determine a final positive or negative result. If the pool again tests as “invalid” the procedure is to proceed directly to testing individual samples. There were no invalid tests on the individual samples.

Details of the processing for the dried blood spots are described in detail elsewhere^7,14^ and summarized here. Total IgG was detected using biotinylated mouse anti-human total IgG (clone H2; Southern Biotech, Birmingham, AL) and IgG4 (clone HP6025; Invitrogen, South San Francisco, CA), and R-phycoerythrin-labeled streptavidin (Invitrogen, South San Francisco, CA). After washing, beads were read on a BioPlex 100 instrument (Bio-Rad, Hercules, CA) using Bio-Plex Manager 6.0 software (Bio-Rad). The level of fluorescence from each sample was reported as the median fluorescence intensity minus background intensity (MFI-BG) where background is the intensity of beads with buffer only. Antibody positivity was determined from a cut-off using ROC analyses^7^. The same bead sets were used for baseline and follow up, with the cut-off value for positivity at 989.

### Data analyses

The mean, standard deviation (SD) and distribution of log MFI-BG at baseline and follow up as well as the individual changes overtime are presented, and the paired t-test was used to test for significant differences between pre and post MDA signals. We define rate as the number of events (sero-conversion or seroreversion) in those at risk per unit of time. Contingency tables were used to compare seroconversion and sero-reversion rates according to the children’s treatment status, the chi-square or fisher’s exact test were used to test for significant differences. Multi-variate logistic/linear regression models, as appropriate, were used to examine the contribution of treatment status and community coverage, age and gender to rates of seroconversion, seroreversion, and change in the LogMFI signal overtime. To account for the clustering of the outcomes at the community level, final models included a random effect for the community.

### Ethical approval

This study was approved by the Johns Hopkins Institutional Review Board and the Tanzania Institute for Medical Research. Written informed consent was obtained from the guardians of each child in the research project, and assent obtained for children over age 7 years. The research followed the tenets of the Declaration of Helsinki.

### Conference presentation

Some of these data were presented at a World Health Organization Trachoma Scientific Informal Workshop in Mozambique, 2019.

## Results

A total of 2,122 children were surveyed at baseline, and 89.9% (1908) were followed after MDA. There was no difference by gender, infection status at baseline, or trachoma status at baseline for those who were not followed up and those in the follow up survey. However children not followed were more likely to be older and to be antibody positive at baseline (Table 1). The primary reasons for 10% loss to follow up was absence from the village during the surveys (88%), refusals (10%), other reasons (2%).

**Table 1:** Baseline characteristics of Children in Longitudinal Cohort by follow-up status.

Characteristic	Followed N = 1908	Not followed N = 214	
Age in years (Mean(SD))	5.4 (2.6)	5.8 (2.7)	**0.014**
Female n (%)	976 (51.2)	98 (46.32)	0.17
TF n (%)	55 (2.9)	8 (3.8)	0.47
TF or TI* n (%)	62 (3.3)	8 (3.8)	0.69
Pgp3 positive n (%)	670 (35.1)	93 (43.5)	**0.016**
*C.T* infection n (%)	120 (6.3)	19 (8.9)	0.15

*Trachomatous inflammation-intense.

At baseline, the TF prevalence in all children in the baseline survey was 3.3% and the prevalence of infection was 6.3%. The Tanzania National Trachoma Control Program was committed to providing MDA to all communities in Kongwa regardless of TF status at this point, affording us the opportunity to observe change in serostatus. The overall coverage in the MDA program for the children in our 50 communities was 65%, but we provided additional resources to ensure our study children received azithromycin, and our final coverage in the children was 97%.

At no survey time were any of the air swab controls positive, supporting an absence of contamination in the field and in the laboratory.

**Figure 1:**
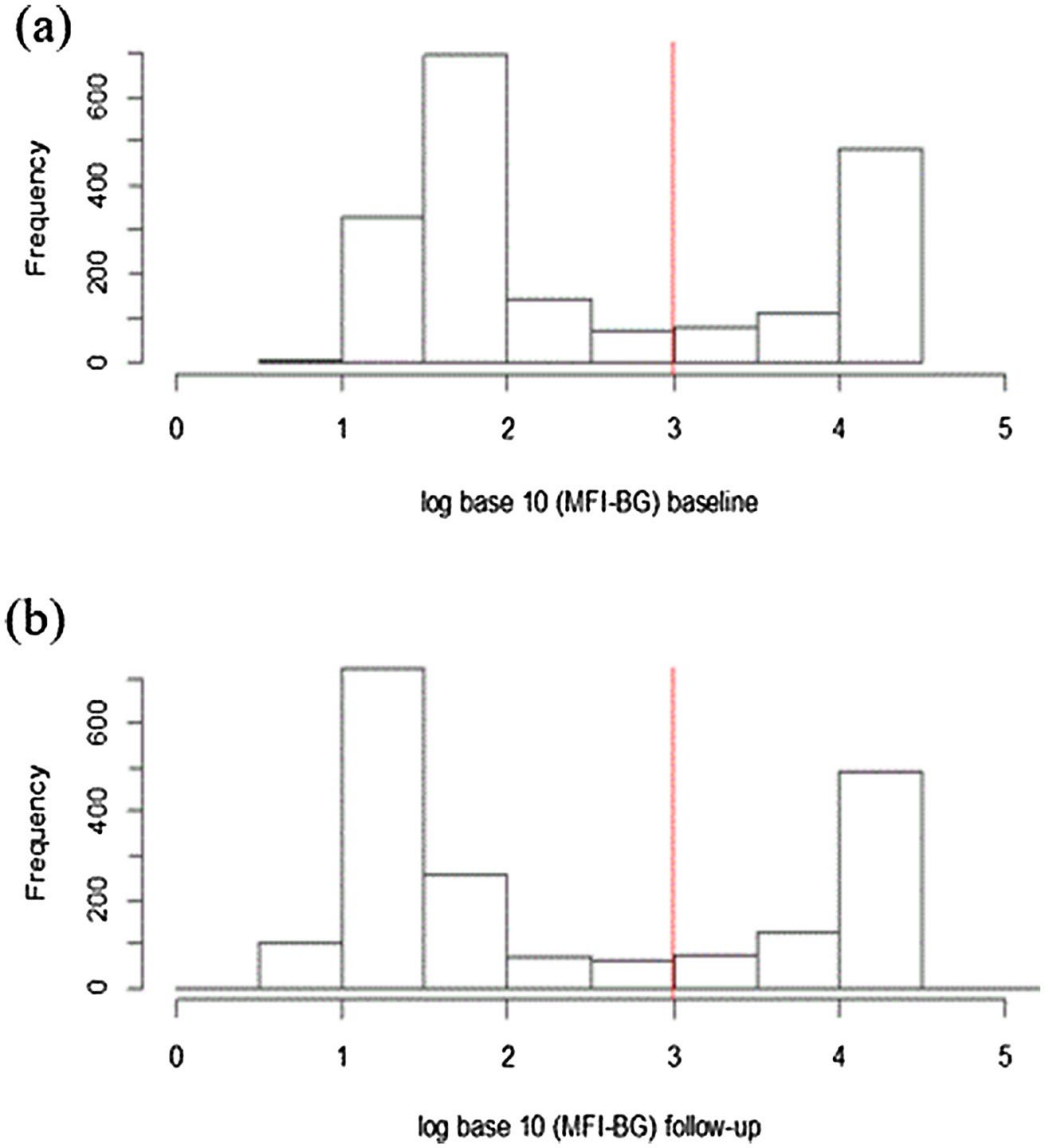
Log_10_ of MFI-BG at baseline (**a**) and at follow up (**b**) for the longitudinal cohort, with the red line showing the cut-off for positivity of each distribution.

**Figure 2:**
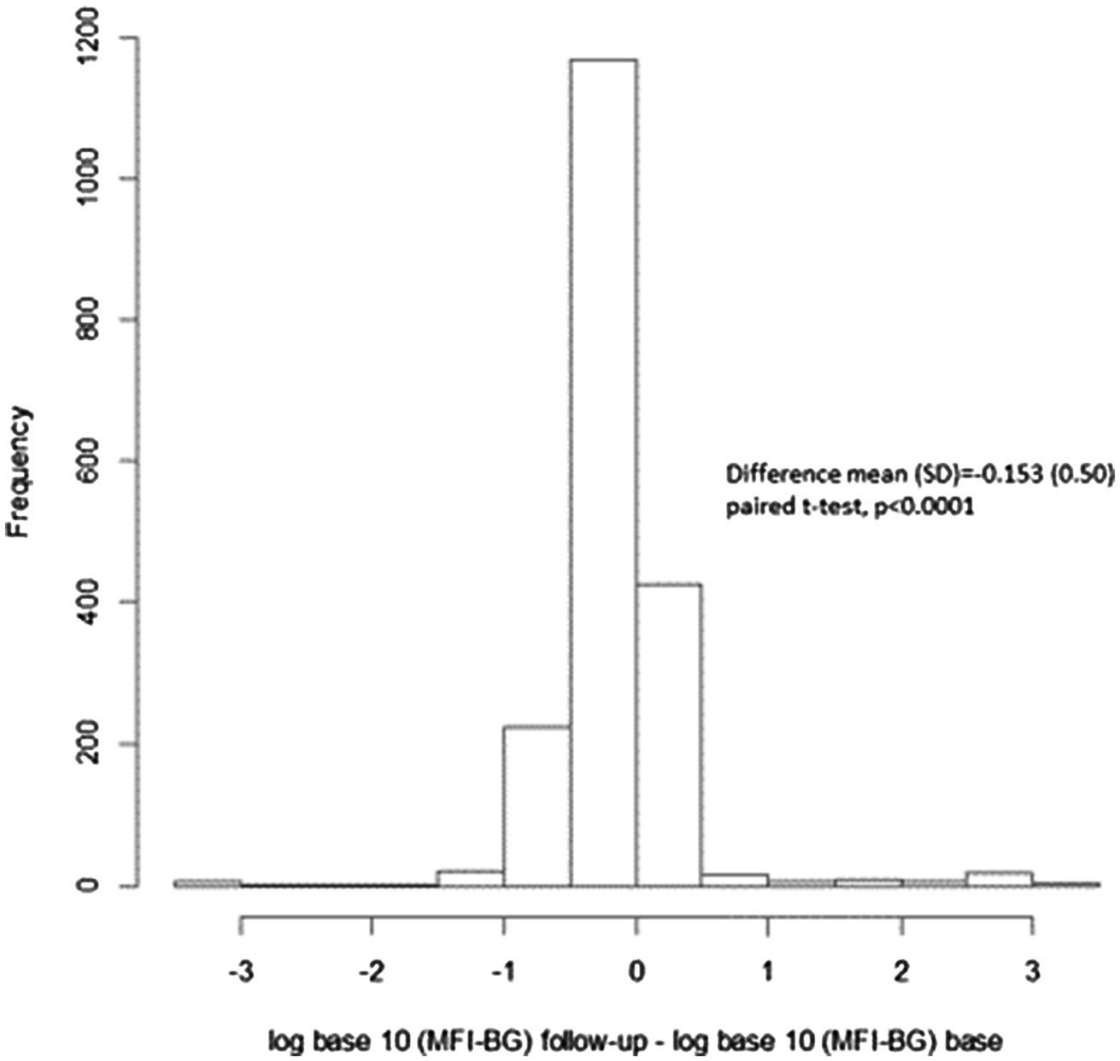
Log_10_MFI-BG at baseline for the longitudinal cohort showing change in mean Log MFI following MDA.

Figure 1 shows the distribution of the log_10_ MFI-BG at baseline and follow up, after MDA suggesting two distributions for each time point. Figure 2 shows the boxplots of change in MFI-BG before and after MDA; the mean (SD) change in the cohort in log MFI-BG was -0.15 (0.50), paired t-test p =  < 0.001, suggesting a small but statistically significant reduction in MFI-BG following MDA.

For the 1908 children in the longitudinal cohort, 670 (35%) were seropositive for antibodies at baseline. Of those who were seropositive at baseline, 27 (4.0%) seroreverted to seronegative after treatment, for an annualized rate of seroreversion of 8%. Of those 1,238 who were negative at baseline, 45 (3.6%) seroconverted to seropositive for an annualized seroconversion rate of 7.2% (Table 2). The loss in log MFI-BG was consistent in every group but the sero-converters, who gained on average 2.09 log MFI-BG.

**Table 2:** Change in log MFI-BG and in antibody status after MDA in children from baseline to 6 months post MDA.

Antibody status	After MDA				Total	Rates (95% confidence intervals) (95%CI)
Baseline	Numbers of SeroNegative	Mean (SD) change in log MFI-BG	Numbers of SeroPositive	Mean (SD) change in log MFI-BG		
Numbers of SeroNegative	1,193	− 0.289 (0.279)	45	2.090 (0.894)	1,238	6 month Sero-conversion rate = 3.6% (95%CI 2.7–4.8)
Numbers of SeroPositive	27	− 1.277 (1.157)	643	− 0.011 (0.172)	670	6 month Sero-reversion rate = 4.0% (95%CI 2.7–6.0)

Coverage was high in the children in our cohort, by design. As Table 3 suggests, there were no sero-reverters in those few who did not receive azithromycin, and sero-conversion rates were higher in that group, but the results were not statistically significant, likely due to the small numbers of children who did not receive MDA. The decline in logMFI-BG was statistically significant for the group that did receive MDA, with virtually no change in the group that did not.

**Table 3:** Comparison of change in logMFI-BG, and serostatus, in children who received MDA and in children who did not.

Received MDA	N	Mean log MFI-BGMFI pre MDA	Mean log MFI-BG post MDA	Difference in mean logMFI-BG	P value	% sero-Reversion	% sero-conversion
Yes	1845	2.57	2.41	− 0.156	< 0.001	27/647 (4.2%)	43/1,198 (3.6%)
No	63	2.68	2.62	− 0.056	0.46	0/23 (0.0%)	2/40 (5.0%)

Table 4 shows the unilateral effect of other factors on change in MFI status, and sero-reversion and sero-conversion rates. The most important factors for loss of MFI was absence of TF or absence of infection in the child at baseline, and no infection or no TF in the community at baseline. Sero-reversion was not seen in children who had infection or TF at baseline, and was highest in communities that had no infection or no TF at baseline. Six of the 120 infected children at baseline were seronegative, and 5 of the 6 had seroconverted by follow up. The one who did not sero-convert despite infection was a 5 year old who did receive MDA and had logMFI-BG value of 1.83 at baseline and 1.38 at follow up.

**Table 4:** Other factors and the association with difference in mean logMFI-BG, seroreversion and seroconversion.

Factor	N	Difference in mean logMFI-BG	P value	Rate of seroreversion	P value	Rate of seroconversion	P value
**Age**							
≤ 5	992	− 0.17	0.17	10/232 (4.3)	0.79	28/760 (3.7)	0.91
6–10	916	− 0.14		17/438 (3.9)		17/478 (3.6)	
**Gender**							
Male	932	− 0.15	0.94	12/304 (4.0)	0.92	23/628 (3.7)	0.96
Female	976	− 0.15		15/366 (4.1)		22/610 (3.6)	
**Had TF at baseline**							
Yes	55	− 0.01	0.01	0/44 (0.0)	0.25	0/11 (0.0)	1.00
No	1853	− 0.16		27/626 (4.3)		45/1,227 (3.7)	
**Had infection at baseline**							
Yes	120	− 0.08	< 0.001	0/114 (0.0)	0.008	5/6 (83.3)	< 0.001
No	1788	− 0.17		27/556 (4.9)		40/1,232 (3.3%)	
**Community**^**a**^** prevalence of TF at baseline**							
0%	848	− 0.18	0.02	18/240 (7.5)	0.001	25/608 (4.1)	0.44
> 0%	1,060	− 0.13		9/430 (2.1)		20/630 (3.2)	
**Community**^**a**^** prevalence of infection at baseline**							
0%	315	− 0.20	0.04	4/77 (5.9)	0.54	7/238 (2.9)	0.70
> 0%	1593	− 0.14		23/593 (3.9)		38/1,000 (3.8)	

^a^Community is defined as the aggregate result from the children in the cohort living in that community.

A multivariate model predicting a loss of at least 0.15 logMFI-BG found that loss was less likely with older age, and less likely if the child had infection at baseline (Table 5). The other factors, presence of TF and infection in the community and if the child had TF at baseline, were also associated with less risk of loss of logMFI-BG, but were not statistically significant once adjusted for infection in the child at baseline. We constructed a model to predict risk of sero-reversion following MDA (Table 6). We could not include individual values of not taking MDA, having infection or TF as sero reversion did not happen in any of those groups. Sero-reversion is less likely in older children, and in children residing in communities where TF is > 0%, particularly > 5%.

**Table 5:** Multivariate model predicting loss of logMFI-BG ≥ 0.15.

Characteristic	Crude association		Multivariate association	
	Odds ratio (95% CI)	p-value	Odds ratio(95% CI)	p-value
Age (per year increase)	0.89 (0.86–0.92)	< 0.0001	0.87 (0.84–0.90)	< 0.0001
Female	0.89 (0.75–1.07)	0.22	0.93 (0.77–1.12)	0.45
TF at baseline	0.27 (0.14–0.52)	< 0.0001	0.53 (0.24–1.14)	0.11
Infection at baseline	0.10 (0.06–0.18)	< 0.0001	0.11 (0.06–0.20)	< 0.001
Community Prevalence of TF at baseline > 0%	0.75 ( 0.63–0.90)	0.002	0.79 (0.56–1.12)	0.18
Community Prevalence of infection at baseline > 0%	0.79 (0.62–1.00)	0.054	0.88 (0.56–1.38)	0.57

**Table 6:** Parsimonious multivariate model predicting seroreversion in a cohort of children following MDA.

Characteristic	Crude association		Multivariate association^a^	
	Odds ratio (95% CI)	p-value	Odds ratio(95% CI)	p-value
Age (per year increase)	0.86 (0.74–1.00)	0.053	0.75 (0.60–0.93)	0.011
**Community prevalence of TF at baseline**				
0%	1.00			
1–5%	0.41 (0.17–0.95)	0.04	0.28 (0.08–0.94)	0.04
> 5%	0.07 (0.01–0.52)	0.001	0.06 (0.01–0.63)	0.034

^a^Model accounts for clustering within community, and adjusts for baseline value of log MFI.

## Discussion

This large cohort study of children in a low trachoma endemic district were followed for changes in their antibody status following MDA. Overall, there was a statistically significant decline in logMFI-BG following MDA, but the change was minor, 0.15 logunits. Within the entire sample, 92% maintained their baseline seropositive/seronegative status after one year, with slightly less seroconversion rates and slightly higher seroreversion rates compared to the previous year in the absence of MDA. This suggest some stability of antibody status at least over one year following MDA in low trachoma endemic communities. The fact there was a decline in logMFI-BG is similar to the findings of Goodhew and colleagues where, 6 months following MDA in a hyperendemic community, the mean MFI-BG declined by 9%^9^; however, in that study there was a small sample of children (n = 105). They reported no sero-reversion whereas we found a rate of seroreversion of 4%. This difference is likely to reflect the different endemicities for trachoma in the two studies, as in our study the prevalence at baseline of TF was < 5% compared to 47% in the hyperendemic community.

In our previous study of this cohort in the absence of MDA, we found a sero-reversion rate of 6%/year, which was also associated with residence in communities with low rates of TF ^8^. There was a slightly lower rate of seroreversion in these children during the year with no MDA, 6%, and what we observed in the year which included MDA, a 6 month rate of 4% ( or a projected annualized rate of 8%), suggesting that decreasing exposure to infection through MDA can result in loss of seropositivity. However, there are limitations in annualizing our observed 6 month rate because of assumptions about the consistency of the rate over the next six months. We might hypothesize that if infection was to re-emerge following MDA, it would be likely greater towards the latter part of the year than closer to the time of MDA; this might result in less sero-reversion in the latter part of the year and more seroconversion. Ideally, we would have data at one year following MDA, but this was not possible as a second MDA was planned by the national program to follow after our survey.

The rate of seroconversion was low in this cohort, an annualized rate of about 7.2%. This rate is less than observed during the year in which there was no MDA, 9.8% (95% CI 8.3–11.4%). MDA has a pronounced effect on infection and disease in treated communities^5,15^ so we would expect exposure to infection to decrease and seroconversion to be low. Seroconversion rates were higher in children who did not have MDA, compared to those who did, but because there were so few who did not have MDA, the difference was not statistically significant. Interestingly, we found a case of a child with infection at baseline who did not sero-convert, which provides further support for the suggestion that multiple infections may be needed to effect sero-conversion. With only two time points in which data were collected on infection and disease, before and after MDA, we do not have sufficient information to determine the number of infections to cause sero-conversion. We also do not know at what time in the course of the 6 months seroconversion or seroreversion occurred. As five of the six infections at baseline sero-converted, there was clearly an effect prior to MDA. However, 40 of the seroconversions we observed were in seronegative children at baseline, and all but 2 had MDA; some of these may have seroconverted prior to MDA, but some may also have seroconverted in the interval from MDA to our follow up survey.

There was loss to follow up, about 10% of children. They tended to be older, and as we have shown previously, older children are more likely to be sero-positive and to maintain seropositivity over time^8^. No other characteristics differentiated the study participants from those lost to follow up, and coupled with the low rate of loss, there is unlikely to be bias in our estimates of the effect of MDA on antibody status.

The rates of seroconversion and seroreversion are unlikely to be due to our choice of cut-off value for positivity because the same bead set was used for all blood spot analyses, and as the data show, the separation into two distributions is quite pronounced. As we have shown previously, the use of other approaches in our data to set cut points had virtually no effect on sero conversion or reversion rates^8^. Nevertheless, as was found previously, we cannot exclude some change in seropositivity status due to fluctuation around the cut-off point.

In summary, MDA has an effect of lowering the MFI in children, but the effect is very modest. Slightly higher rates of sero-reversion were observed in this cohort compared to rates observed in the cohort in the absence of MDA. The yearly rate of seroconversion to antibody positive declined following MDA, from 9.8%/year to 7.2%, as expected with decreased exposure to infection. Rates of change in antibody status in this cohort are consistent with decreasing exposure to infection as a result of MDA.

## Data Availability

All de-identified data are available to qualified researchers through a materials transfer agreement.
